# qPCRtools: An R package for qPCR data processing and visualization

**DOI:** 10.3389/fgene.2022.1002704

**Published:** 2022-09-13

**Authors:** Xiang Li, Yingmin Wang, Jingyu Li, Xinyue Mei, Yixiang Liu, Huichuan Huang

**Affiliations:** ^1^ State Key Laboratory for Conservation and Utilization of Bio-Resources in Yunnan, Yunnan Agri-cultural University, Kunming, China; ^2^ Key Laboratory for Agro-Biodiversity and Pest Control of Ministry of Education, Yunnan Agricultural University, Kunming, China

**Keywords:** bioinformatics, qPCR, R package, visualization, gene expression

## Abstract

In biological research, qPCR is a technique that is frequently used to measure gene expression levels. The calculation of gene amplification efficiency is a critical step in the processing of qPCR data since it helps to decide which method to employ to compute gene expression levels. Here, we introduce the R package qPCRtools, which enables users to analyze the efficiency of gene amplification. Additionally, this software can determine gene expression levels using one of three approaches: the conventional curve-based method, the 2^−ΔΔCt^ method, and the SATQPCR method. The qPCRtools package produces a table with the statistical data of each method as well as a figure with a box or bar plot illustrating the results. The R package qPCRtools is freely available at CRAN (https://CRAN.R-project.org/package=qPCRtools) or GitHub (https://github.com/lixiang117423/qPCRtools/tree/main/CRAN/qPCRtools).

## 1 Introduction

Quantitative PCR (qPCR) is a method for the precise quantification of gene expression. The experimental procedure for qPCR includes sample/template preparation, assay optimization/validation, RT‒PCR, and data analysis (Bustin et al., 2010). For data analysis, several methods can be chosen, such as the most widely used 2^−ΔΔCT^ method. The 2^−ΔΔCt^ method is a convenient way to analyze the relative changes in gene expression from qPCR experiments. However, it assumes an almost equal amplification efficiency for both the internal target gene and the reference gene (Livak and Schmittgen, 2001). If the amplification efficiency does not meet the prerequisites of the 2^−ΔΔCT^ method, absolute quantification should be used (Whelan et al., 2003; Dhanasekaran et al., 2010). Due to the limitations of absolute quantification, such as its time-consuming nature, relative quantification is preferred. In relative quantification, in which the expression of a target gene is measured in relation to one or multiple reference genes, the results can be considerably influenced by various factors. A major point that must be considered when using a relative quantification approach is the amplification efficiency. It has been shown that even minor variations in amplification efficiency can lead to considerable variation in the calculated gene expression value (Pfaffl, 2001). One way of calculating the amplification efficiency is to produce serial dilutions of the target genes. Then, the Ct values are plotted on a logarithmic scale along with the corresponding concentrations. Next, a linear regression curve based on the data points is generated, and the slope of the trend line is calculated. Finally, efficiency is calculated using the following equation: E = −1+10(−1/slope) (the dilution factor is 10). The amplification efficiency value can help us choose the best method for processing qPCR data. On the other hand, the relative standard curve can be used to handle qPCR data. The data analyses required in qPCR experiments, including the calculation of primer amplification efficiency, gene expression levels, and final statistics, are relatively difficult for novices. Many tools have been developed to process qPCR data (Pabinger et al., 2014). However, data wrangling is required to apply some of these tools (Mar et al., 2009; Lievens et al., 2012; Rödiger et al., 2015), and the web services of some tools, such as CampER (Blom et al., 2020) and PCR-Miner (Zhao and Fernald, 2005), are no longer available.

To address these limitations, we have developed qPCRtools, a package developed based on the statistical computing language R and the widely used R visualization package ggplot2 (Wickham, 2016), for processing qPCR data. The main features of qPCRtools include the following: 1) construction of relative standard curves from gradient dilution qPCR data and calculation of the amplification efficiency (for any dilution factor); 2) calculation of the gene expression based on several methods, including the relative standard curve method, 2^−ΔΔCT^ method (Livak and Schmittgen, 2001), and RqPCR method (Rancurel et al., 2019); and 3) statistical calculations based on the *t* test or ANOVA with Tukey’s test. All results generated from all functions except CalRTable include a table, detailed results with statistical information, and a figure in which the table results can be visualized.

## 2 Description

### 2.1 Overview of the qPCRtools package

The qPCRtools package was created based on the R language (R Core Team, 2013). Five functions are included in the qPCRtools package for the processing and display of qPCR data (Table 1). The amount of RNA needed for reverse transcription can be determined using the CalRTable function. The CalCurve function can determine the amplification efficiency of each gene, in addition to calculating the relative standard curve for each gene based on equal dilution. The expression levels of each gene can be determined using the other three functions in various ways.

**TABLE 1:** This table shows all features of qPCRtools.

Function	Description
CalRTable	Calculates volume for reverse transcription
CalCurve	Calculates a relative standard curve
CalExpCurve	Calculates expression values using the relative standard curve method
CalExp2ddCt	Calculates expression using the 2^−ΔΔCt^ method
CalExpRqPCR	Calculates expression values using the RqPCR method

### 2.2 Case study

#### 2.2.1 Example 1: Calculation of RNA volume for reverse transcription

Reverse transcription will be necessary to generate cDNA for qPCR after RNA extraction. The user can obtain the reagent volume table from the protocol provided in the kit. However, when the average RNA concentration is being determined for a large number of samples, it will take some time to generate the final table. Reverse transcription can be performed by creating a table of each component’s volume using the CalRTable function. As an example, we consider the EasyScript All-in-One First-Strand cDNA Synthesis SuperMix for qPCR (One-Step gDNA Removal) (TransGen Biotech, AE341) reverse transcription reagent kit, which only comprises two components: SuperMix and gDNA Remover. In this case, we will require 4 μl of SuperMix and 1 μl of gDNA remover for 1 μg of total RNA. To determine the volume of total RNA, its concentration must be known. To obtain the average concentration, three or more replications of each sample are needed. The volumes of RNA and other components can be estimated using the CalRTable function (Table 2). There must be a column “all” in the second input file as shown in the example data.

**TABLE 2:** Example of CalRTable output.

Sample	Average concentration	RNA (μl)	SuperMix (μl)	gDNARemover (μl)	H_2_O (μl)	Total (μl)
1	160.40	12.47	8.00	2.00	17.53	40.00
2	163.33	12.24	8.00	2.00	17.76	40.00
3	182.57	10.95	8.00	2.00	19.05	40.00
4	203.80	9.81	8.00	2.00	20.19	40.00
5	180.13	11.10	8.00	2.00	18.90	40.00
6	171.83	11.64	8.00	2.00	18.36	40.00

#### 2.2.2 Example 2: Calculation of the relative standard curve and amplification efficiency

**FIGURE 1:**
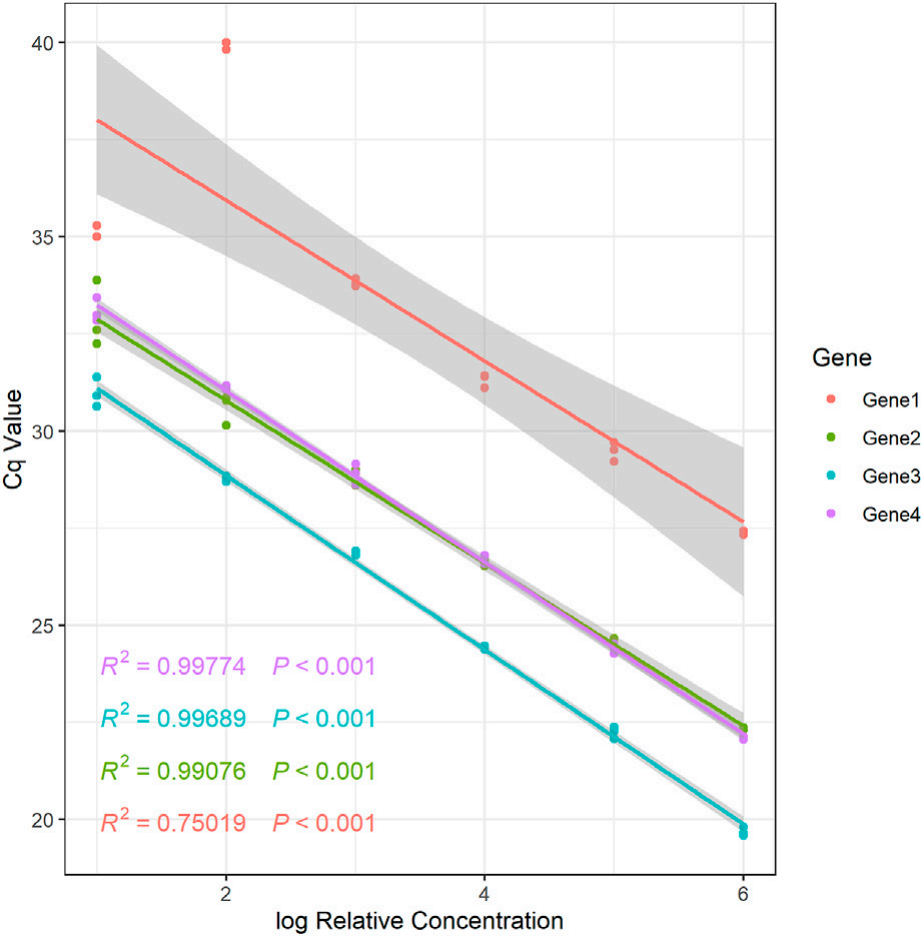
The relative standard curve of example genes.

For qPCR studies as well as for final data processing and analysis, it is essential to known the primer amplification efficiency (Livak and Schmittgen, 2001; Pfaffl, 2001), since it determines which technique should be applied to analyze qPCR data. For novel primers, the initial stage is the evaluation of amplification efficiency. The CalCurve function can be used to determine the amplification efficiency based on the concentration gradient dilution approach (Supplementary Table S1). Additionally, the function provides a regression equation for relative concentrations that have been log-transformed and the accompanying Cq values (Figure 1 and Supplementary Table S1). The relative expression levels of genes under various treatments can be determined using the regression equation.

#### 2.2.3 Example 3: Calculation of gene expression levels using the relative standard curve method

**FIGURE 2:**
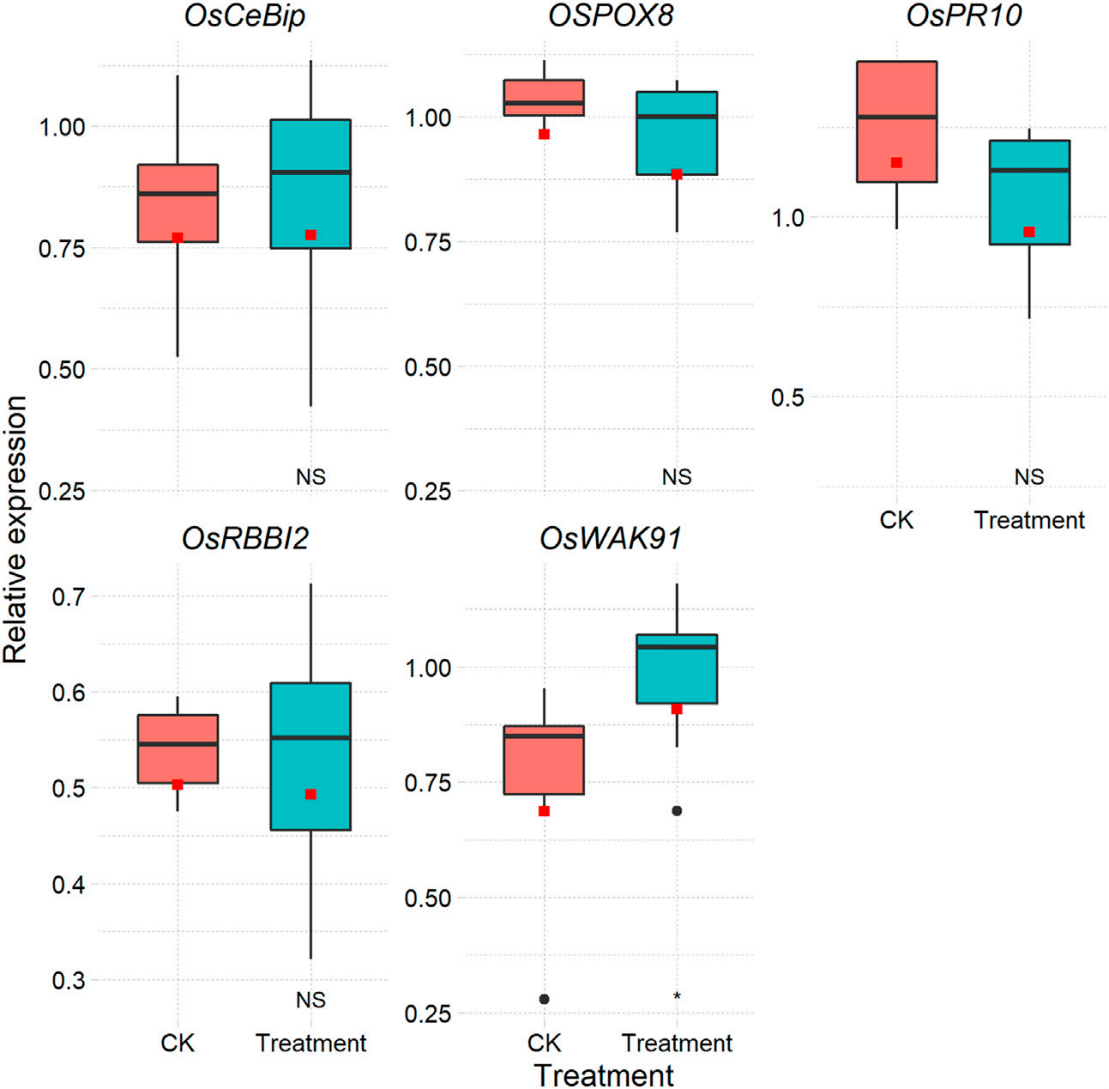
Box plot of each gene in different treatments based on the relative standard curve method.

After the above step, if the amplification efficiencies of reference genes and target genes are not equal, the 2^−ΔΔCt^ method cannot be used (Livak and Schmittgen, 2001). Some methods without a reference gene can also be chosen, such as the method based on the Markov chain Monte Carlo algorithm (Matz et al., 2013). One way to determine the expression level that disregards amplification efficiency is the relative standard curve approach. Based on the relative standard curve depicted above, the CalExpCurve function can calculate the expression levels of individual genes. A table (Supplementary Table S2) and a figure are included in the results (Figure 2).

#### 2.2.4 Example 4: Calculation of expression levels using the 2^−ΔΔCt^ method

**FIGURE 3:**
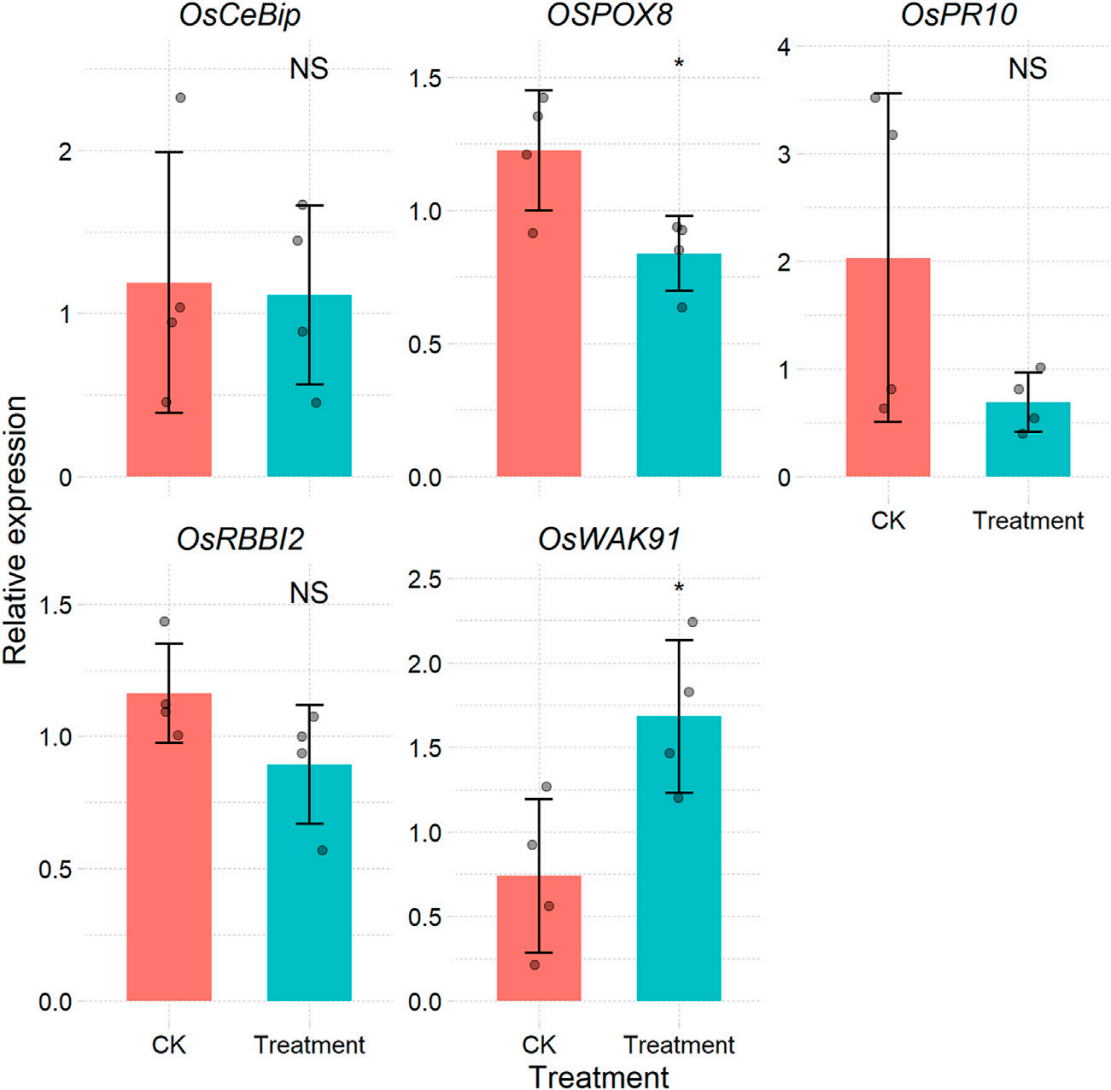
Bar plot of the relative expression of each gene under different treatments calculated via the 2^−ΔΔCt^ method.

If the amplification efficiency of the target genes is consistent with that of the reference gene, the most widely used2^−ΔΔCt^ method is available for expression level calculation (Livak and Schmittgen, 2001). There are many software packages that can apply this method, such as the R package pcr (Ahmed and Kim, 2018). However, most of these packages require some data wrangling before their application. For users who have large samples and data, this approach is time-consuming and is not reproducible. The CalExp2ddCt function can process raw Cq data from the qPCR machine and then produces a table (Supplementary Table S3) and figure (Figure 3) illustrating the expression levels. More importantly, the templates can be reused.

#### 2.2.5 Example 5: Calculation of expression levels using the RqPCR method

**FIGURE 4:**
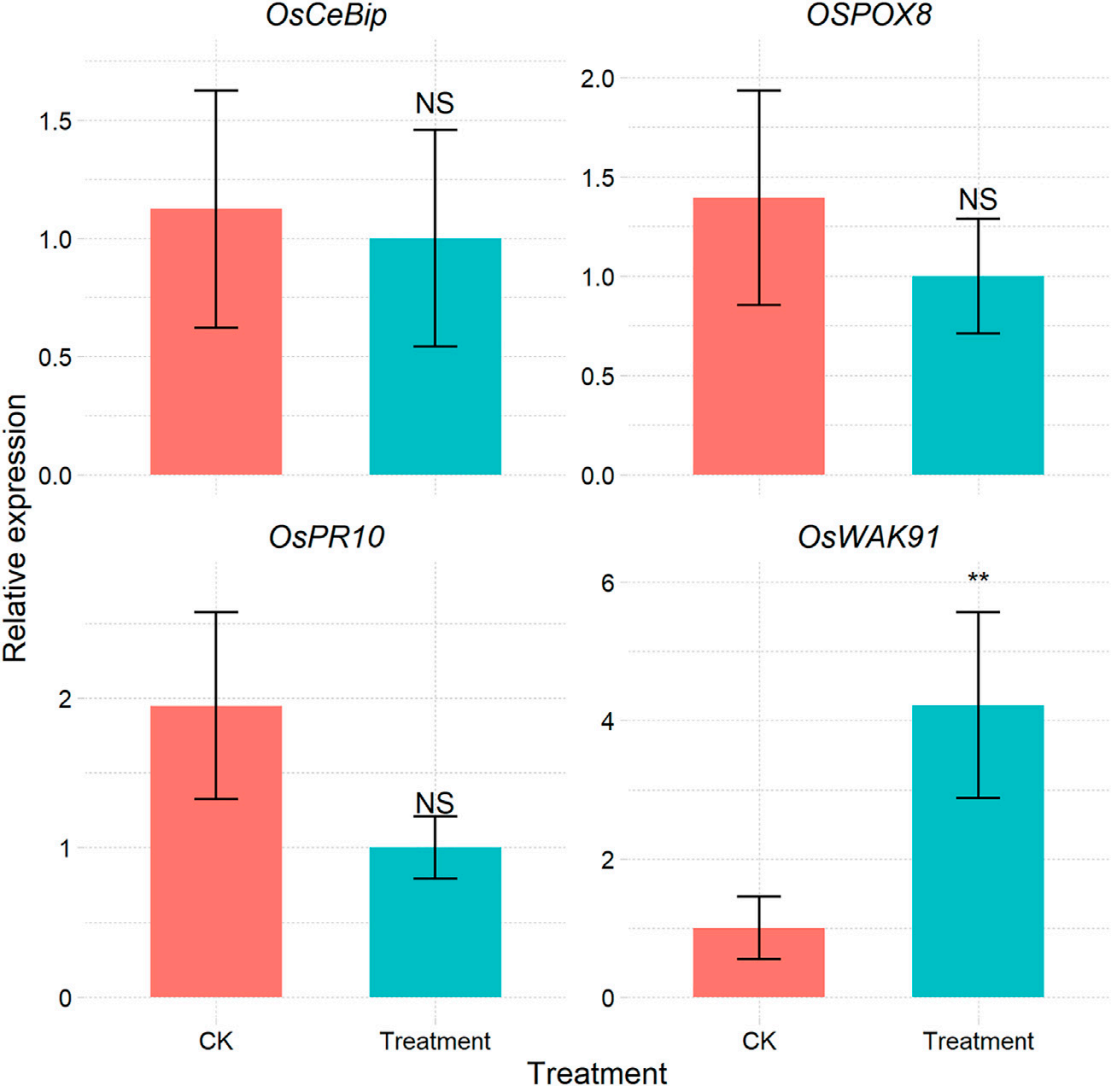
Bar plot of the relative expression of each gene under different treatments calculated via the RqPCR method.

For most methods used in qPCR data processing, a reference is required to correct the expression values of the target genes (Livak and Schmittgen, 2001). Some methods do not require reference genes; such methods are based on the Markov chain Monte Carlo algorithm (Matz et al., 2013) or SATQPCR (Rancurel et al., 2019) according to the MIQE guidelines (dMIQE Group and Huggett, 2020). The CalExpRqPCR function can implement this function from SATQPCR (Supplementary Table S4 and Figure 4), and the required amplification efficiency can be calculated using the CalCurve function.

## 3 Conclusion

An important step in qPCR is calculating the efficiency of gene primer amplification, but previous R packages cannot perform this step effectively. Here, we offer qPCRtools, a fully functional tool that can not only quickly determine the amplification efficiency of gene primers but also calculate gene expression levels from a Cq table based on several methods that return statistical results and visualization figures. The resulting figures are ggplot objects that can be further customized by applying scale and theme settings and superimposed annotation layers.

## Data Availability

The original contributions presented in the study are included in the article/Supplementary Material; further inquiries can be directed to the corresponding author.

## References

[B1] Ahmed M. Kim D. R. (2018). pcr: an R package for quality assessment, analysis and testing of qPCR data. PeerJ 6, e4473. 10.7717/peerj.4473 29576953 PMC5858653

[B2] Blom J. Rückert C. Kalinowski J. Goesmann A. (2020). CAmpER–a software for the calculation of amplification efficiencies for real-time PCR-experiments; 2007. Available at: https://www.gene-quantification.de/qpcr2007/publications/Blom-qPCR-2007.pdf.

[B3] Bustin S. A. Beaulieu J.-F. Huggett J. Jaggi R. Kibenge F. S. B. Olsvik P. A. (2010). MIQE précis: Practical implementation of minimum standard guidelines for fluorescence-based quantitative real-time PCR experiments. BMC Mol. Biol. 11, 74. 10.1186/1471-2199-11-74 20858237 PMC2955025

[B4] Dhanasekaran S. Doherty T. M. Kenneth J. (2010). Comparison of different standards for real-time PCR-based absolute quantification. J. Immunol. Methods 354, 34–39. 10.1016/j.jim.2010.01.004 20109462

[B5] dMIQE Group Huggett J. F. (2020). The digital MIQE guidelines update: Minimum information for publication of quantitative digital PCR experiments for 2020. Clin. Chem. 66, 1012–1029. 10.1093/clinchem/hvaa125 32746458

[B6] Lievens A. Van Aelst S. Van Den Bulcke M. Goetghebeur E. (2012). Enhanced analysis of real-time PCR data by using a variable efficiency model: FPK-PCR. Nucleic Acids Res. 40, e10. 10.1093/nar/gkr775 22102586 PMC3258155

[B7] Livak K. J. Schmittgen T. D. (2001). Analysis of relative gene expression data using real-time quantitative PCR and the 2(-Delta Delta C(T)) Method. Methods 25, 402–408. 10.1006/meth.2001.1262 11846609

[B8] Mar J. C. Kimura Y. Schroder K. Irvine K. M. Hayashizaki Y. Suzuki H. (2009). Data-driven normalization strategies for high-throughput quantitative RT-PCR. BMC Bioinforma. 10, 110. 10.1186/1471-2105-10-110 PMC2680405 19374774

[B9] Matz M. V. Wright R. M. Scott J. G. (2013). No control genes required: Bayesian analysis of qRT-PCR data. PLOS ONE 8, e71448. 10.1371/journal.pone.0071448 23977043 PMC3747227

[B10] Pabinger S. Rödiger S. Kriegner A. Vierlinger K. Weinhäusel A. (2014). A survey of tools for the analysis of quantitative PCR (qPCR) data. Biomol. Detect. Quantif. 1, 23–33. 10.1016/j.bdq.2014.08.002 27920994 PMC5129434

[B11] Pfaffl M. W. (2001). A new mathematical model for relative quantification in real-time RT–PCR. Nucleic Acids Res. 29, e45. 10.1093/nar/29.9.e45 11328886 PMC55695

[B12] Rancurel C. Van Tran T. Elie C. Hilliou F. (2019). Satqpcr: Website for statistical analysis of real-time quantitative PCR data. Mol. Cell. Probes 46, 101418. 10.1016/j.mcp.2019.07.001 31283967

[B17] R Core Team (2013). R: A language and environment for statistical computing.

[B13] Rödiger S. Burdukiewicz M. Schierack P. (2015). chipPCR: an R package to pre-process raw data of amplification curves. Bioinformatics 31, 2900–2902. 10.1093/bioinformatics/btv205 25913204

[B14] Whelan J. A. Russell N. B. Whelan M. A. (2003). A method for the absolute quantification of cDNA using real-time PCR. J. Immunol. Methods 278, 261–269. 10.1016/s0022-1759(03)00223-0 12957413

[B15] Wickham H. (2016). ggplot2: elegant graphics for data analysis. Springer.

[B16] Zhao S. Fernald R. D. (2005). Comprehensive algorithm for quantitative real-time polymerase chain reaction. J. Comput. Biol. 12, 1047–1064. 10.1089/cmb.2005.12.1047 16241897 PMC2716216

